# Ductal Margin Distance Is a Stronger Prognostic Indicator than Margin Status After Curative Resection of Distal Cholangiocarcinoma

**DOI:** 10.3390/cancers18132165

**Published:** 2026-07-06

**Authors:** Yeongsoo Jo, Yoo-Seok Yoon, Ho-Seong Han, Jai Young Cho, Jun Suh Lee, Boram Lee

**Affiliations:** 1 Department of Surgery, Ewha Womans University Seoul Hospital, Ewha Womans University College of Medicine, Seoul 07804, Republic of Korea; dudtn87411@gmail.com; 2 Department of Surgery, Seoul National University Bundang Hospital, Seoul National University College of Medicine, Seongnam-si 13620, Republic of Korea; hanhs@snubh.org (H.-S.H.); jychogs@gmail.com (J.Y.C.); boramlee0827@snubh.org (B.L.); 3 Department of Surgery, Bucheon Sejong Hospital, Bucheon-si 14754, Republic of Korea; rudestock@gmail.com

**Keywords:** high-grade dysplasia, carcinoma in situ, ductal margin distance, ductal margin status, extrahepatic cholangiocarcinoma

## Abstract

Background: Distal cholangiocarcinoma (dCCA) has a poor prognosis and the prognostic impact of ductal margin status, particularly carcinoma in situ (CIS) or high-grade dysplasia (HGD) at the margin, remains controversial. This retrospective single-center study evaluated whether ductal margin distance (DMD) is a stronger prognostic indicator than ductal margin status (DMS) after curative resection. Methods: We reviewed 416 patients who underwent pancreaticoduodenectomy for distal cholangiocarcinoma between 2003 and 2022. DMS was classified as negative, CIS/HGD, or invasive carcinoma (IC). The DMD was defined pathologically as the linear distance from the proximal ductal margin to IC and patients were divided into two groups using 10 mm as the cut-off. Survival was analyzed with Kaplan–Meier and Cox regression. Results: Overall, 349, 52, and 15 patients had negative, CIS/HGD, and IC margins, and 249 and 167 had DMD > 10 and ≤10 mm, respectively. Overall survival (OS) and disease-free survival (DFS) did not differ significantly according to DMS, whereas DMD ≤ 10 mm was associated with significantly worse 5-year OS (48.5% vs. 67.9%) and DFS (39.9% vs. 55.9%). On multivariable analysis, DMD ≤ 10 mm was an independent adverse prognostic factor for both OS (hazard ratio 1.669; *p* = 0.003) and DFS (hazard ratio 1.394; *p* = 0.026). Conclusions: DMD demonstrated a stronger association with prognosis than DMS in patients with negative or CIS/HGD at the ductal resection margin following dCCA resection. A DMD of ≤10 mm was significantly associated with decreased OS and DFS. Well-designed prospective studies are warranted to validate these results.

## Simple Summary

Distal cholangiocarcinoma often recurs even after curative surgery, and accurate prognostic markers are needed to guide postoperative surveillance and treatment. Margin status of the bile duct is routinely reported, but the clinical significance of carcinoma in situ or high-grade dysplasia at the margin remains debated. In this study, we examined whether the distance from the proximal bile duct margin to invasive cancer on pathology provides better prognostic information than margin status alone. We analyzed 416 patients who underwent pancreaticoduodenectomy for distal cholangiocarcinoma and compared outcomes by margin status and by a 10 mm cut-off for margin-to-tumor distance. Margin status was not clearly associated with survival, whereas a distance of 10 mm or less was linked to substantially worse overall and recurrence-free survival, even after accounting for other factors. These findings suggest that reporting and using margin-to-tumor distance may improve risk stratification after surgery and support more tailored follow-up and adjuvant treatment strategies.

## 1. Introduction

The incidence of distal cholangiocarcinoma (dCCA) has steadily increased over recent decades [1,2], and its overall prognosis remains poor [3,4]. Complete surgical resection is currently the only treatment modality with the potential for long-term survival in patients with dCCA [5,6,7]. Among the established prognostic factors, ductal margin status (DMS) has been recognized as a key determinant of oncologic outcomes [8,9,10,11], and positive ductal margins are associated with unfavorable survival outcomes [6,12,13,14,15,16,17,18,19,20,21,22,23]. However, achieving a negative ductal resection margin is technically challenging because of the tumor’s characteristic longitudinal spread along the biliary tract [24,25]. Furthermore, additional resections, including hilar bile duct or major liver resection, may be necessary to obtain a negative margin but substantially increase the risks of postoperative morbidity and mortality.

Recent studies have reported conflicting results regarding the prognostic relevance of DMS, particularly in patients with carcinoma in situ (CIS)/high-grade dysplasia (HGD) at the ductal resection margin. Wakai et al. demonstrated that the long-term survival of patients with remnant CIS/HGD at the ductal margin was comparable to that of patients with histologically negative ductal resection margins following dCCA resection [26]. Subsequent studies yielded similar results, suggesting that the presence of remnant CIS/HGD does not have a negative effect on long-term survival after curative resection [27,28,29,30,31,32,33]. In contrast, several recent studies have reported that remnant CIS/HGD at the ductal margin has a negative impact on recurrence and survival, particularly in patients with relatively early-stage dCCA [34,35,36].

Most prior studies assessed the impact of the DMS, based solely on the histologic presence of CIS/HGD, without considering the actual distance between the invasive carcinoma (IC) and the ductal margin. We hypothesized that a longer ductal margin distance (DMD) would be associated with better oncologic outcomes and that its prognostic significance might depend more on the proximity of the margin to the invasive carcinoma than on the existence of CIS/HGD. Therefore, this study investigated the prognostic significance of the DMD in patients with negative DMS, including those with CIS/HGD at the ductal resection margin, following surgical resection of dCCA.

## 2. Materials and Methods

### 2.1. Study Population and Design

For this single-center retrospective study, we reviewed the clinical data of patients who underwent surgical resection for dCCA between June 2003 and December 2022 at Seoul National University Bundang Hospital, South Korea. A total of 493 consecutive patients were identified. After excluding 81 patients who underwent palliative surgery (*n* = 32), hepatopancreaticoduodenectomy (*n* = 2), or bile duct resection (*n* = 43), this study included 416 patients who underwent pancreaticoduodenectomy (PD). The location of the bile duct cancer was classified based on the epicenter of the gross tumor, using the origin of the cystic duct as a reference landmark. We compared the oncologic outcomes, including the recurrence and survival rates, according to the DMS and DMD, and analyzed the prognostic factors for survival.

This study was conducted in accordance with the ethical principles of the Declaration of Helsinki and approved by the hospital’s Institutional Review Board (approval no. B-2307-840-104). The requirement for informed consent was waived owing to the retrospective nature of the study.

### 2.2. Pathological Assessment

The extrahepatic bile duct in the resected specimen was opened longitudinally from the ampulla of Vater up to the proximal resection margin (PRM) to accurately assess the DMS and DMD. The PRM was evaluated on permanent formalin-fixed sections. The DMS was classified into three categories: negative, positive with CIS/HGD, and positive with IC. R1 resection was defined as the presence of IC at the ductal resection margin or radial margin. The DMD was defined as the linear distance between the PRM and the proximal end of the IC. It was assessed microscopically on permanent glass slides along the longitudinal ductal axis of the opened bile duct specimen during routine histopathologic evaluation. Depending on the time period of diagnosis, one or two board-certified pathologists were involved in case assessment, and any discrepant interpretations were resolved through a consensus discussion. To minimize the potential impact of sampling, the specimens were serially sectioned at 5 mm intervals, and the entire proximal bile duct margin in each case was submitted for formalin-fixed paraffin-embedded tissue blocks. Using the maximal χ^2^ method in conjunction with the Cox proportional hazards model, we identified 10 mm as the optimal DMD cut-off for survival and recurrence and divided the patients into two groups: DMD > 10 mm and DMD ≤ 10 mm. The 10 mm threshold was therefore a data-driven cut-off derived from the present cohort, rather than a prespecified value based on prior clinical or pathological evidence.

During the study period, pathological staging was performed according to the AJCC edition in use at the time of diagnosis. Because the definition of pathological T stage changed across AJCC editions, uniform retrospective reclassification of T stage according to a single edition was not feasible without complete re-review of all original pathological slides. Therefore, AJCC 7th and 8th edition T stages were summarized separately for descriptive comparison. In addition, numerical maximal tumor diameter was extracted from the pathology records and analyzed as an objective continuous variable to account, at least in part, for tumor burden and local tumor extent.

### 2.3. Statistical Analysis

All data were analyzed using SPSS version 22.0 for Windows (SPSS, Chicago, IL, USA). Continuous variables were compared using Student’s *t* test or the Mann–Whitney U test, as appropriate, and are reported as the mean ± standard deviation or median with interquartile range according to their distribution. For comparisons involving more than two groups, one-way analysis of variance or the Kruskal–Wallis test was used, as appropriate. Pairwise post hoc comparisons were performed using the Mann–Whitney U test with Holm correction. Categorical variables were compared using the χ^2^ test or Fisher’s exact test. Univariate and multivariable analyses were performed using the Cox proportional hazard model to identify independent predictors of overall survival (OS) and disease-free survival (DFS). Furthermore, the maximal χ^2^ method and Cox proportional hazard model were used simultaneously to calculate the DMD cut-off value. Multivariable Cox regression models were constructed to account for potential confounding. In addition to variables showing statistical significance in univariate analysis, clinically relevant covariates were also considered a priori, including age, preoperative CA19-9 level, preoperative biliary drainage, maximal tumor diameter, lymph node metastasis, histologic grade, radial margin status, major vessel invasion, lymphovascular invasion, perineural invasion, adjuvant chemotherapy, adjuvant radiotherapy, and ductal margin distance. Postoperative survival was calculated using the Kaplan–Meier method, and differences in the survival curves were compared using the log-rank test. In all tests, a *p* value of <0.05 was regarded as statistically significant. To assess the robustness of the 10 mm threshold, we additionally analyzed DMD as a continuous variable in the Cox proportional hazards model using the same covariates as in the main multivariable model. We also performed restricted cubic spline analyses to explore the shape of the association between DMD and survival outcomes and to evaluate potential non-linearity.

## 3. Results

### 3.1. Patient Characteristics According to the DMS and DMD

Table 1 and Table 2 summarize the patient characteristics according to the DMS and DMD. Based on the DMS classification, the negative, CIS/HGD, and IC groups comprised 349, 52, and 15 patients, respectively. Maximal tumor diameter differed significantly among the three DMS groups. The IC group had larger tumors than both the negative and CIS/HGD groups, whereas maximal tumor diameter was comparable between the negative and CIS/HGD groups (median, 35.0 mm vs. 28.0 mm and 28.0 mm; *p* = 0.005). The proportions of patients who received adjuvant chemotherapy (*p* = 0.003) and adjuvant radiotherapy (*p* < 0.001) were significantly greater in the IC group than in the other groups. However, there were no significant differences in sex, age, preoperative cancer antigen 19-9 levels, preoperative biliary drainage, lymph node metastasis, histologic grade, major vessel invasion, lymphovascular invasion, and perineural invasion (Table 1). According to the DMD classification, the >10 mm group comprised 249 patients and the ≤10 mm group comprised 167 patients. Maximal tumor diameter was significantly larger in the DMD ≤ 10 mm group than in the DMD > 10 mm group (median, 34.0 mm vs. 25.0 mm; *p* < 0.001). Adjuvant radiotherapy was significantly more frequent in the ≤10 mm group (*p* = 0.029); there were no significant differences in the other clinicopathologic characteristics (Table 2).

### 3.2. Survival Outcomes According to the DMS

**Figure 1:**
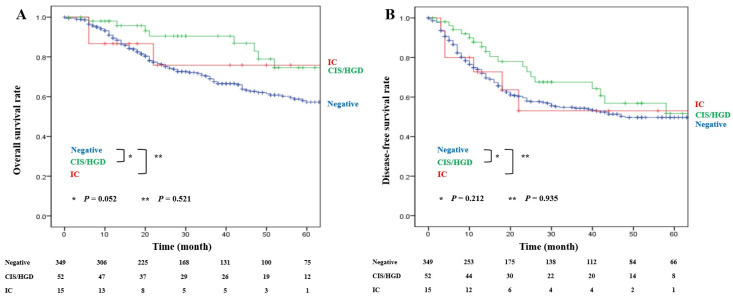
Survival outcomes according to the ductal margin status: (**A**) overall survival; (**B**) disease-free survival.

OS and DFS according to the DMS are presented in Figure 1. The 1-, 3-, and 5-year OS rates were 91.0%, 70.4%, and 58.0%, respectively, in the negative group; 98.0%, 90.5%, and 74.6%, respectively, in the CIS/HGD group; and 86.7%, 75.8%, and 75.8%, respectively, in the IC group (Figure 1A). There were no significant differences in the OS rates according to the DMS among the three groups (negative vs. CIS/HGD, *p* = 0.052; negative vs. IC, *p* = 0.521).

The 1-, 3-, and 5-year DFS rates were 74.9%, 54.8%, and 49.7%, respectively, in the negative group; 87.8%, 67.6%, and 51.7%, respectively, in the CIS/HGD group; and 72.7%, 53.0%, and 53.0%, respectively, in the IC group (Figure 1B). There were no significant differences in the DFS rates according to the DMS (negative vs. CIS/HGD, *p* = 0.212; negative vs. IC, *p* = 0.935).

### 3.3. Survival Outcomes According to the DMD

**Figure 2:**
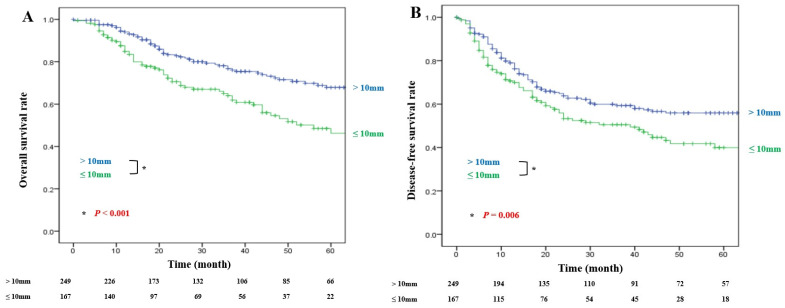
Survival outcomes according to the ductal margin distance: (**A**) overall survival; (**B**) disease-free survival.

OS and DFS according to the DMD are presented in Figure 2. The OS and DFS rates were significantly lower in patients with DMD ≤ 10 mm than in those with DMD > 10 mm. The 1-, 3-, and 5-year OS rates were 94.5%, 78.1%, and 67.9%, respectively, in the DMD > 10 mm group; and 87.6%, 65.0%, and 48.5%, respectively, in the DMD ≤ 10 mm group (*p* < 0.001) (Figure 2A). The 1-, 3-, and 5-year DFS rates were 79.9%, 59.9%, and 55.9%, respectively, in the DMD > 10 mm group; and 71.3%, 50.5%, and 39.9%, respectively, in the DMD ≤ 10 mm group (*p* = 0.006) (Figure 2B).

### 3.4. Survival Outcomes According to the DMD Stratified by the DMS and Lymph Node Metastasis

**Figure 3:**
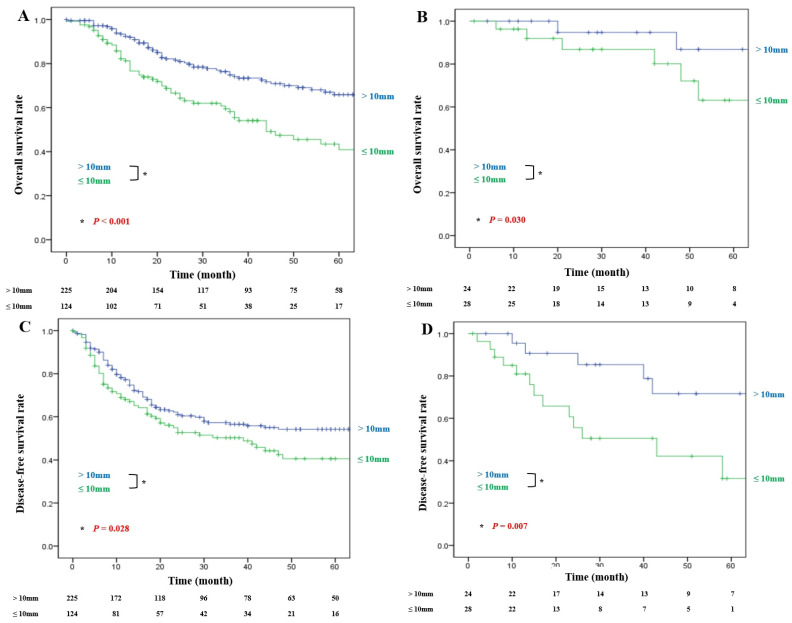
Survival outcomes according to the ductal margin distance: (**A**) overall survival in the negative ductal margin; (**B**) overall survival in the CIS/HGD at the margin; (**C**) disease-free survival in the negative ductal margin; (**D**) disease-free survival in the CIS/HGD at the margin.

The subgroup analyses according to the DMS and lymph node metastasis revealed consistent findings. In patients with negative ductal margins, the OS rates were significantly lower in the DMD ≤ 10 mm group than in the DMD > 10 mm group (Figure 3A; 1-year: 93.9% vs. 85.8%; 3-year: 76.3% vs. 59.5%; 5-year: 65.9% vs. 43.4%; *p* < 0.001). In patients with CIS/HGD at the margin, the OS rates were significantly lower in the DMD ≤ 10 mm group than in the DMD > 10 mm group (Figure 3B; 1-year: 100.0% vs. 96.3%; 3-year: 94.7% vs. 86.8%; 5-year: 86.8% vs. 63.1%; *p* = 0.030). In both the negative and CIS/HGD subgroups, the DFS rates were also significantly lower in the DMD ≤ 10 mm group than in the DMD > 10 mm group (Figure 3C; 1-year: 78.2% vs. 69.0%; 3-year: 57.3% vs. 50.3%; 5-year: 54.3% vs. 40.6%; *p* = 0.028) (Figure 3D; 1-year: 95.5% vs. 81.0%; 3-year: 85.3% vs. 50.6%; 5-year: 71.6% vs. 31.6%; *p* = 0.007).

### 3.5. CIS Carcinoma In Situ, HGD High-Grade Dysplasia

In patients without lymph node metastasis, the OS (Supplementary Figure S1A; 1-year, 95.6% vs. 93.6%; 3-year: 83.2% vs. 73.3%; 5-year: 74.7% vs. 60.8%; *p* = 0.016) and DFS (Supplementary Figure S1B; 1-year: 86.1% vs. 80.4%; 3-year: 70.2% vs. 61.9%; 5-year: 66.4% vs. 50.6%; *p* = 0.034) rates were lower in the DMD ≤ 10 mm group than in the DMD > 10 mm group. In patients with lymph node metastasis, the OS rates were significantly lower in the DMD ≤ 10 mm group than in the DMD > 10 mm group (Supplementary Figure S1C; 1-year: 92.3% vs. 79.3%; 3-year: 66.0% vs. 53.5%; 5-year: 49.7% vs. 30.8%; *p* = 0.025). Although the DFS rates tended to be lower in the DMD ≤ 10 mm group, the difference did not reach statistical significance (Supplementary Figure S1D; 1-year: 67.7% vs. 59.1%; 3-year: 38.6% vs. 34.4%; 5-year: 34.4% vs. 24.9%; *p* = 0.216).

### 3.6. Prognostic Factors for Survival

In univariate analysis, maximal tumor diameter was significantly associated with OS (HR 1.013 per 1 mm increase; 95% CI, 1.002–1.024; *p* = 0.021). However, it was not retained as an independent prognostic factor in multivariable analysis. The multivariable analysis identified five independent predictors of OS in the entire cohort: age > 70 years (hazard ratio [HR] 1.710; *p* = 0.002), positive lymph node metastasis (N[+]; HR 1.644; *p* = 0.007), lymphovascular invasion (HR 1.959; *p* < 0.001), perineural invasion (HR 1.755; *p* = 0.010), and DMD ≤ 10 mm (HR 1.669; *p* = 0.003) (Table 3).

In univariate analysis, maximal tumor diameter was also significantly associated with DFS (HR 1.011 per 1 mm increase; 95% CI, 1.001–1.020; *p* = 0.024), but it was not retained as an independent prognostic factor in multivariable analysis. Lymph node metastasis (HR 1.819; *p* < 0.001), histologic grade (moderate differentiation: HR 1.784; *p* = 0.049; poor differentiation: HR 2.606; *p* = 0.004), major vessel invasion (HR 1.697; *p* = 0.041), lymphovascular invasion (HR 1.550; *p* = 0.006), and DMD ≤ 10 mm (HR 1.394; *p* = 0.026) were identified as independent adverse prognostic factors for DFS (Table 4). To address potential model-selection bias and the possible confounding effects of adjuvant therapy and tumor burden, we additionally fitted expanded multivariable Cox models that included clinically relevant covariates regardless of their univariate significance, including maximal tumor diameter, adjuvant chemotherapy, and adjuvant radiotherapy. In these expanded models, maximal tumor diameter was not independently associated with OS or DFS, whereas DMD ≤ 10 mm remained independently associated with worse OS (HR 1.550; *p* = 0.013) and DFS (HR 1.363; *p* = 0.043), supporting the robustness of the main findings.

In additional sensitivity analyses, DMD remained significantly associated with both OS and DFS when modeled as a continuous variable. In the multivariable Cox model, each 1 mm increase in DMD was associated with a lower risk of death (HR 0.986, *p* = 0.009) and recurrence (HR 0.988, *p* = 0.011). Restricted cubic spline analyses also demonstrated a significant overall association between shorter DMD and worse OS (overall *p* = 0.030) and DFS (overall *p* = 0.021), without statistically significant evidence of non-linearity (*p* = 0.322 and *p* = 0.196, respectively). These findings support the robustness of the prognostic association beyond simple dichotomization at 10 mm.

## 4. Discussion

Previous studies that evaluated the prognostic impact of CIS/HGD in patients with resected extrahepatic cholangiocarcinoma have examined the prognostic role of the DMS, but reported inconsistent results [26,27,28,29,30,31,32,33,34,35,36,37,38,39,40,41]. However, none of those studies investigated the prognostic relevance of the DMD. In this study, we examined the long-term oncologic outcomes of patients who underwent curative-intent surgical resection of dCCA according to their DMS and DMD. Our findings indicate that DMD showed a more consistent association with prognosis than DMS in this cohort of patients with resected dCCA. However, because the number of patients with IC-positive ductal margins was small, these results should not be interpreted as evidence that DMS has no prognostic relevance.

A shorter DMD (≤10 mm) was significantly associated with worse OS across the entire cohort and in subgroups stratified by margin status and lymph node metastasis. In contrast, there were no significant differences in the OS and DFS rates among the negative, CIS/HGD, and IC groups according to the DMS. Furthermore, the multivariable analysis revealed that the DMD, but not the DMS, was an independent prognostic factor for OS, and additional sensitivity analyses using DMD as a continuous variable yielded consistent associations for both OS and DFS. Although maximal tumor diameter was associated with both OS and DFS in univariate analyses, it was not retained as an independent prognostic factor in the multivariable models. In contrast, DMD ≤ 10 mm remained independently associated with adverse outcomes, suggesting that DMD may provide prognostic information beyond tumor size alone. These findings suggest that the DMD should be considered when interpreting the prognostic implications of CIS/HGD, which remains a controversial issue. One possible explanation for this finding is that the DMD may capture both the biological extent of tumor spread and the adequacy of surgical clearance more accurately than histologic classification alone. Distal cholangiocarcinoma is characterized by longitudinal extension along the biliary epithelium, and the simple presence or absence of CIS/HGD at the ductal margin may not fully reflect how close the invasive component is to the resection line. In contrast, the DMD provides a quantitative measure of the residual distance between the invasive tumor front and the proximal ductal margin. Therefore, a shorter DMD may indicate either more extensive subclinical longitudinal tumor spread or a narrower oncologic clearance, both of which could contribute to a higher risk of recurrence and worse survival. From this perspective, DMD may offer more integrated prognostic information than DMS alone, especially in patients with negative margins or CIS/HGD at the ductal margin. Although the 10 mm cut-off was derived using the maximal χ^2^ method, additional analyses treating DMD as a continuous variable showed consistent results for both OS and DFS. Furthermore, spline-based modeling confirmed an overall inverse association between DMD and adverse outcomes, without evidence of marked non-linearity. Although the 10 mm cut-off was derived using the maximal χ^2^ method, additional analyses treating DMD as a continuous variable showed consistent results for both OS and DFS. Furthermore, spline-based modeling confirmed an overall inverse association between DMD and adverse outcomes, without evidence of marked non-linearity. Because this threshold was derived entirely from the present dataset and was not based on a prespecified biological or pathological boundary, it should be interpreted as a clinically useful prognostic stratification point rather than an absolute biological cut-off. External validation in independent cohorts is required before this threshold can be generalized or incorporated into routine clinical practice. Clinically, these findings may help surgeons assess the need for additional resection and the appropriate extent of surgery, including hilar bile duct or major liver resection, and may also help refine postoperative treatment strategies when CIS/HGD is detected on intraoperative frozen biopsy or postoperative pathology.

All subgroup analyses demonstrated significant differences in the OS and DFS rates according to the DMD, thereby supporting its role as a strong prognostic indicator, with the exception of DFS in patients with lymph node metastasis. Numerous studies have shown that lymph node metastasis is one of the strongest prognostic factors in patients with resected dCCA [28,41,42,43]. Accordingly, the dominant influence of nodal metastasis on recurrence likely attenuated the statistical impact of the DMD in this subgroup. Biologically, this may reflect the fact that DMD primarily represents the extent of local longitudinal tumor spread and the adequacy of local ductal clearance, whereas lymph node metastasis may indicate a more advanced systemic tumor phenotype with a higher propensity for early recurrence. In node-positive disease, the risk of recurrence may therefore be driven more strongly by nodal tumor burden than by the residual distance between the invasive tumor and the ductal margin. This may explain why the prognostic effect of DMD on DFS was attenuated in this subgroup, even though the overall direction of the association remained similar. Nonetheless, patients with DMD > 10 mm exhibited a trend toward improved DFS among those with lymph node metastasis, despite the absence of statistical significance. We also recognize that variable selection based solely on univariate significance may introduce model bias. Therefore, we performed additional multivariable analyses including clinically relevant covariates irrespective of their univariate significance, and the prognostic impact of DMD remained significant. This supports the robustness of our main findings against potential model-specification bias.

This study has several limitations. First, its retrospective single-center design and the long inclusion period (2003–2022) may have introduced temporal bias. During this interval, surgical practice, perioperative management, pathologic assessment, and adjuvant treatment strategies likely evolved. Improvements in operative technique and postoperative care may have influenced long-term survival outcomes independently of the ductal margin variables. In addition, changes in pathologic processing or interpretation over time may have affected the assessment of ductal margin status and the measurement of ductal margin distance. Likewise, the indications and regimens for adjuvant therapy were not uniform throughout the study period, which may also have contributed to survival heterogeneity. Detailed regimen-level information could not be consistently reconstructed for all patients because of the retrospective design and long study period. Therefore, our findings should be interpreted with caution, and external validation in more contemporary, standardized cohorts is warranted.

Second, the number of patients with invasive carcinoma (IC) at the ductal margin was very small (*n* = 15), which substantially limited the statistical power to detect survival differences according to DMS. Therefore, the absence of a significant association between DMS and OS or DFS in our cohort should not be interpreted as evidence of no prognostic effect. Rather, these negative findings likely reflect imprecision related to the very small IC subgroup, as suggested by the wide confidence intervals. Thus, our results indicate that DMD provided more consistent prognostic discrimination in this dataset, but they do not exclude a potential prognostic role of DMS, particularly for IC-positive margins. Although the DMS has traditionally been regarded as a key prognostic factor in patients with resected dCCA, several studies of patients undergoing pancreaticoduodenectomy have reported that the DMS alone was not a significant predictor of survival [44,45,46,47,48]. Moreover, adjuvant radiotherapy or chemoradiotherapy was shown to improve survival outcomes in patients with resected extrahepatic cholangiocarcinoma, particularly those with R1 margins [49,50,51]. Therefore, it is plausible that postoperative radiotherapy attenuated the survival difference between the IC group and the negative or CIS/HGD groups in our cohort. Because adjuvant radiotherapy was more frequently administered in patients with unfavorable margin characteristics, we considered it a potential confounder in the survival analyses. However, additional multivariable models adjusting explicitly for adjuvant radiotherapy still showed a significant adverse prognostic effect of DMD ≤ 10 mm. These findings suggest that the association between shorter DMD and poorer outcomes was not solely attributable to imbalances in postoperative radiotherapy use. In this context, our analysis of the DMD, a quantitative index of the extent of the resection margin, provides meaningful insight beyond conventional assessments based on the margin status.

Third, uniform reassessment of pathological T stage according to a single AJCC edition was not feasible because the study period spanned multiple AJCC staging systems and complete re-review of all original pathological slides was not available. To address this limitation, we summarized pathological T stage separately according to the AJCC edition used at the time of diagnosis and additionally incorporated numerical maximal tumor diameter from the pathology records into the clinicopathologic and survival analyses. Maximal tumor diameter was significantly larger in the DMD ≤ 10 mm group than in the DMD > 10 mm group and was associated with both OS and DFS in univariate Cox analyses. However, it was not retained as an independent prognostic factor in the multivariable models, whereas DMD ≤ 10 mm remained independently associated with adverse outcomes. These findings suggest that the prognostic value of DMD is not solely explained by maximal tumor diameter. Nevertheless, maximal tumor diameter cannot fully substitute for pathological tumor depth or uniformly reassessed T stage; therefore, residual confounding by local tumor extent cannot be completely excluded.

Despite these limitations, this study, to the best of our knowledge, is the first to identify the prognostic role of the DMD for predicting recurrence and survival after surgical resection of dCCA. These findings may have clinical implications for intraoperative and postoperative treatment planning, particularly in patients with CIS/HGD at the ductal resection margin. However, because DMD is measured on permanent pathologic sections, it is not directly available during surgery and cannot be used as a real-time intraoperative metric. Rather, its value may lie in informing the interpretation of intraoperative frozen-biopsy findings and in refining the conceptual framework for surgical decision-making. For example, when CIS/HGD is identified at the proximal ductal margin, the surgeon must often judge whether further resection is warranted based on the estimated extent of proximal ductal spread, the gross resection length already achieved, and the operative risk of additional surgery. In this context, our findings suggest that a sufficiently wide ductal clearance may be more relevant than histologic classification alone, although prospective studies are needed to determine whether surrogate intraoperative indicators can reliably approximate postoperative DMD.

Although obtaining a negative resection margin should remain the surgical goal, our findings should not be interpreted as definitive evidence to omit additional resection in patients with CIS/HGD at the ductal margin. Rather, in selected patients—particularly elderly individuals or those with substantial comorbidities—a DMD > 10 mm may serve as an adjunctive factor in individualized intraoperative decision-making when the potential benefits of further resection must be weighed against its operative risks. Furthermore, because standardized criteria for adjuvant therapy in dCCA have yet to be established, our findings suggest that patients with DMD ≤ 10 mm may warrant closer postoperative risk stratification and consideration of adjuvant treatment; however, these implications should be validated in prospective studies before being incorporated into routine practice. In the era of increasingly personalized cancer therapy, refined postoperative risk stratification may become more relevant for tailoring surveillance and adjuvant treatment strategies. In this broader context, recent advances in biologically informed cancer therapeutics further underscore the importance of moving beyond conventional clinicopathologic classifications alone.

## 5. Conclusions

In conclusion, DMD provided more consistent prognostic stratification than DMS in patients with negative margins or CIS/HGD at the ductal resection margin after resection of dCCA. A DMD ≤ 10 mm was significantly associated with worse OS and DFS, supporting the potential value of DMD as a postoperative prognostic marker. However, because the IC-positive margin subgroup was small, these findings should not be interpreted as excluding the prognostic relevance of DMS. Further studies are needed to determine whether intraoperative surrogate indicators can reliably approximate postoperative DMD and inform surgical decision-making.

## Figures and Tables

**Table 1:** Characteristics of patients classified according to the ductal margin status.

Variables	Negative (*N* = 349)	CIS/HGD (*N* = 52)	IC (*N* = 15)	*p* Value
Sex (male)	220 (63.0%)	35 (67.3%)	12 (80.0%)	0.202
Age (years) > 70	152 (43.6%)	28 (53.8%)	7 (46.7%)	0.307
Preoperative CA19-9 > 37 U/mL	192 (55.0%)	32 (61.5%)	10 (66.7%)	0.259
Preoperative biliary drainage	295 (84.5%)	44 (84.6%)	14 (93.3%)	0.572
AJCC 7th edition T stage (*N* = 198)	*N* = 174	*N* = 21	*N* = 3	0.676
7th T1	13 (7.5%)	0 (0.0%)	0 (0.0%)	
7th T2	54 (31.0%)	7 (33.3%)	3 (100.0%)	
7th T3	101 (58.0%)	14 (66.7%)	0 (0.0%)	
7th T4	6 (3.4%)	0 (0.0%)	0 (0.0%)	
AJCC 8th edition T stage (*N* = 218)	*N* = 175	*N* = 31	*N* = 12	0.240
8th T1	48 (27.4%)	11 (35.5%)	3 (25.0%)	
8th T2	101 (57.7%)	20 (64.5%)	8 (66.7%)	
8th T3	25 (14.3%)	0 (0.0%)	1 (8.3%)	
8th T4	1 (0.6%)	0 (0.0%)	0 (0.0%)	
Maximal tumor diameter (mm, median [IQR])	28.0 [22.0–35.5]	28.0 [24.0–36.0]	35.0 [32.0–45.5]	0.005
Lymph node metastasis	131 (37.5%)	18 (34.6%)	8 (53.3%)	0.530
Histologic grade				0.644
Well differentiated	43 (12.3%)	6 (11.5%)	3 (20.0%)	
Moderately differentiated	238 (68.2%)	38 (73.1%)	8 (53.3%)	
Poorly differentiated	51 (14.6%)	8 (15.4%)	3 (20.0%)	
Not reported or others	17 (4.9%)	0 (0.0%)	1 (6.7%)	
Positive radial margin	7 (2.0%)	1 (1.9%)	0 (0.0%)	0.762
Major vessel invasion	21 (6.0%)	4 (7.7%)	1 (6.7%)	0.834
Lymphovascular invasion	153 (43.8%)	23 (44.2%)	9 (60.0%)	0.358
Perineural invasion	249 (69.9%)	34 (65.4%)	13 (86.7%)	0.582
Adjuvant chemotherapy	116 (33.2%)	24 (46.2%)	10 (66.7%)	0.003
Adjuvant radiotherapy	34 (9.7%)	12 (23.1%)	8 (53.3%)	<0.001
Ductal margin distance (≤10 mm)	124 (35.5%)	28 (53.8%)	15 (100.0%)	<0.001

CIS, carcinoma in situ, HGD, high-grade dysplasia, IC, invasive carcinoma, CA19-9, cancer antigen 19-9, IQR, interquartile range. AJCC T stage was summarized separately according to the edition used at the time of pathological diagnosis because the definitions differed between editions.

**Table 2:** Characteristics of patients classified according to the ductal margin distance.

Variables	>10 mm (*N* = 249)	≤10 mm (*N* = 167)	*p* Value
Sex (male)	159 (63.9%)	108 (64.7%)	0.865
Age (years) > 70	112 (45.0%)	75 (44.9%)	0.989
Preoperative CA19-9 > 37 U/mL	132 (53.0%)	102 (61.1%)	0.104
Preoperative biliary drainage	209 (83.9%)	144 (86.2%)	0.523
AJCC 7th edition T stage (*N* = 198)	*N* = 122	*N* = 76	0.582
7th T1	8 (6.6%)	5 (6.6%)	
7th T2	36 (29.5%)	28 (36.8%)	
7th T3	75 (61.5%)	40 (52.6%)	
7th T4	3 (2.5%)	3 (3.9%)	
AJCC 8th edition T stage (*N* = 218)	*N* = 127	*N* = 91	0.448
8th T1	38 (29.9%)	24 (26.4%)	
8th T2	67 (52.8%)	62 (68.1%)	
8th T3	22 (17.3%)	4 (4.4%)	
8th T4	0 (0.0%)	1 (1.1%)	
Maximal tumor diameter (mm, median [IQR])	25.0 [20.0–35.0]	34.0 [27.0–42.5]	<0.001
Lymph node metastasis	85 (34.1%)	72 (43.1%)	0.064
Histologic grade			0.468
Well differentiated	31 (12.4%)	21 (12.6%)	
Moderately differentiated	167 (67.1%)	117 (70.1%)	
Poorly differentiated	37 (14.9%)	25 (15.0%)	
Not reported or others	14 (5.6%)	4 (2.4%)	
Positive radial margin	4 (1.6%)	4 (2.4%)	0.566
Major vessel invasion	14 (5.6%)	12 (7.2%)	0.519
Lymphovascular invasion	111 (44.6%)	74 (44.3%)	0.957
Perineural invasion	166 (66.7%)	125 (74.9%)	0.074
Adjuvant chemotherapy	93 (37.3%)	57 (34.1%)	0.503
Adjuvant radiotherapy	25 (10.0%)	29 (17.4%)	0.029
Ductal margin status			<0.001
Negative	225 (90.4%)	124 (74.3%)	
CIS/HGD	24 (9.6%)	28 (16.8%)	
IC	0 (0.0%)	15 (9.0%)	

CA19-9, cancer antigen 19-9, CIS, carcinoma in situ, HGD, high-grade dysplasia, IC, invasive carcinoma, IQR, interquartile range. AJCC T stage was summarized separately according to the edition used at the time of pathological diagnosis because the definitions differed between editions.

**Table 3:** Prognostic factors for overall survival in all patients.

	Univariate Analysis			Multivariable Analysis		
Variables	HR	95% CI	*p* Value	HR	95% CI	*p* Value
Sex (male)	1.334	0.933–1.906	0.114			
Age (years) > 70	1.519	1.080–2.137	0.016	1.710	1.211–2.416	0.002
Preoperative CA19-9 > 37 U/mL	1.601	1.126–2.275	0.009			
Preoperative biliary drainage	1.447	0.881–2.377	0.145			
Maximal tumor diameter (mm)	1.013	1.002–1.024	0.021			
Lymph node metastasis	2.122	1.512–2.978	<0.001	1.644	1.149–2.352	0.007
Histologic grade						
Well differentiated	-	-	-			
Moderately differentiated	1.879	1.032–3.423	0.039			
Poorly differentiated	1.776	0.862–3.661	0.120			
Positive radial margin	3.600	1.581–8.196	0.002			
Major vessel invasion	2.133	1.176–3.869	0.013			
Lymphovascular invasion	2.301	1.632–3.245	<0.001	1.959	1.374–2.793	<0.001
Perineural invasion	2.160	1.424–3.277	<0.001	1.755	1.144–2.693	0.010
Adjuvant chemotherapy	0.812	0.560–1.178	0.272			
Adjuvant radiotherapy	0.964	0.587–1.584	0.885			
Ductal margin status						
Negative	-	-	-			
CIS/HGD	0.549	0.296–1.017	0.057			
IC	0.683	0.217–2.150	0.515			
Ductal margin distance ≤ 10 mm	1.828	1.306–2.558	<0.001	1.669	1.189–2.343	0.003

HR, hazard ratio, CI, confidence interval, CA19-9, cancer antigen 19-9, CIS, carcinoma in situ, HGD, high-grade dysplasia, IC, invasive carcinoma.

**Table 4:** Prognostic factors for disease-free survival in all patients.

	Univariate Analysis			Multivariable Analysis		
Variables	HR	95% CI	*p* Value	HR	95% CI	*p* Value
Sex (male)	1.180	0.870–1.600	0.288			
Age (years) > 70	1.024	0.763–1.374	0.875			
Preoperative CA19-9 > 37 U/mL	1.446	1.070–1.954	0.017			
Preoperative biliary drainage	1.816	1.128–2.921	0.014			
Maximal tumor diameter (mm)	1.011	1.001–1.020	0.024			
Lymph node metastasis	2.426	1.809–3.252	<0.001	1.819	1.324–2.500	<0.001
Histologic grade						
Well differentiated	-	-	-	-	-	-
Moderately differentiated	2.283	1.289–4.042	0.005	1.784	1.003–3.174	0.049
Poorly differentiated	3.193	1.684–6.055	<0.001	2.606	1.368–4.966	0.004
Positive radial margin	1.987	0.816–4.840	0.130			
Major vessel invasion	2.704	1.677–4.358	<0.001	1.697	1.021–2.819	0.041
Lymphovascular invasion	2.112	1.573–2.836	<0.001	1.550	1.137–2.114	0.006
Perineural invasion	1.771	1.253–2.503	0.001			
Adjuvant chemotherapy	1.231	0.913–1.661	0.173			
Adjuvant radiotherapy	1.081	0.709–1.649	0.717			
Ductal margin status						
Negative	-	-	-			
CIS/HGD	0.741	0.460–1.193	0.218			
IC	0.976	0.432–2.207	0.954			
Ductal margin distance ≤ 10 mm	1.496	1.117–2.003	0.007	1.394	1.040–1.868	0.026

HR, hazard ratio, CI, confidence interval, CA19-9, cancer antigen 19-9, CIS, carcinoma in situ, HGD, high-grade dysplasia, IC, invasive carcinoma.

## Data Availability

The datasets presented in this article are not readily available because of patient privacy and legal/ethical issues.

## Supplementary Materials

The following supporting information can be downloaded at: https://www.mdpi.com/article/10.3390/cancers18132165/s1, Figure S1. Comparison of (A) overall survival and (B) disease-free survival rates according to the ductal margin distance in patients without lymph node metastasis. Comparison of (C) overall survival and (D) disease-free survival rates according to the ductal margin distance in patients with lymph node metastasis.
