# Supplementary material for: Ductal Margin Distance Is a Stronger Prognostic Indicator than Margin Status After Curative Resection of Distal Cholangiocarcinoma
Source: Cancers (Basel). 2026 Jul 6;18(13):2165. doi: 10.3390/cancers18132165 (PMC13360584; doi:10.3390/cancers18132165)
Supplement: Supplementary file 1 [file cancers-18-02165-s001.zip › cancers-4388816-supplementary.pdf]

SUPPLEMENTARY FIGURE 1A

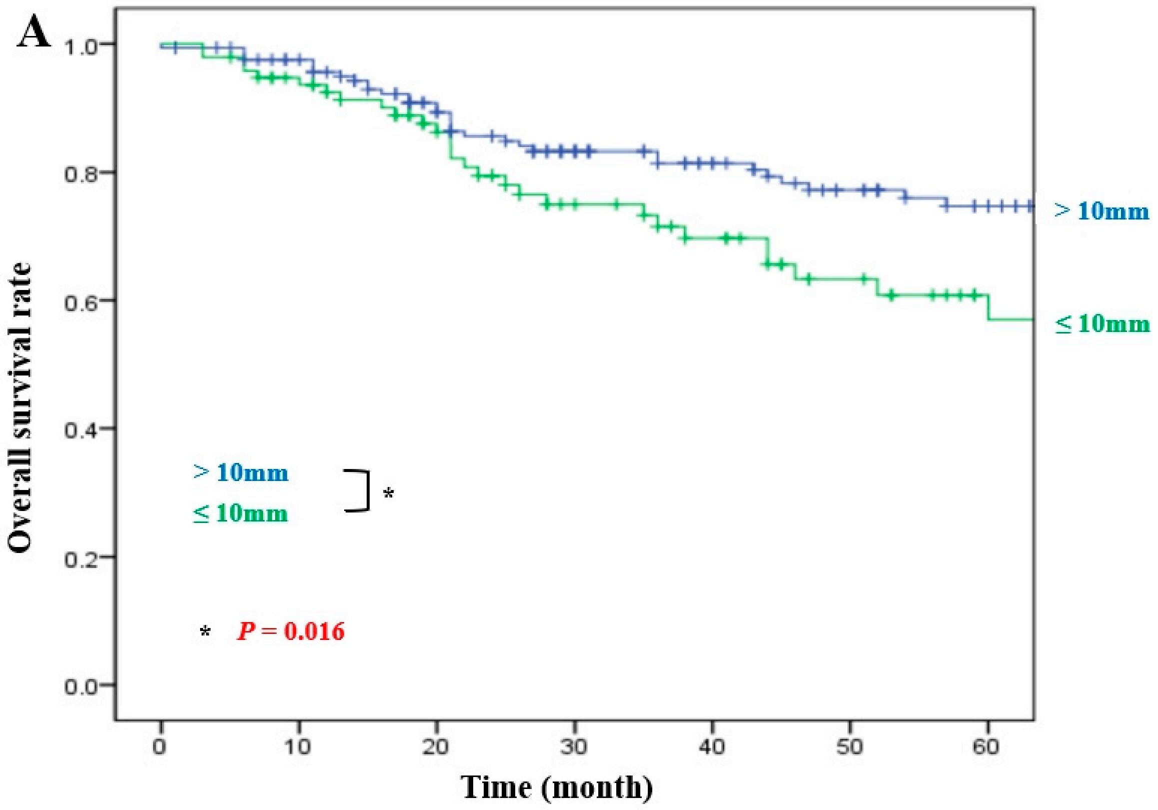

> 10mm	164	151	124	99	83	68	56
≤ 10mm	95	85	66	46	37	26	16

SUPPLEMENTARY FIGURE 1B

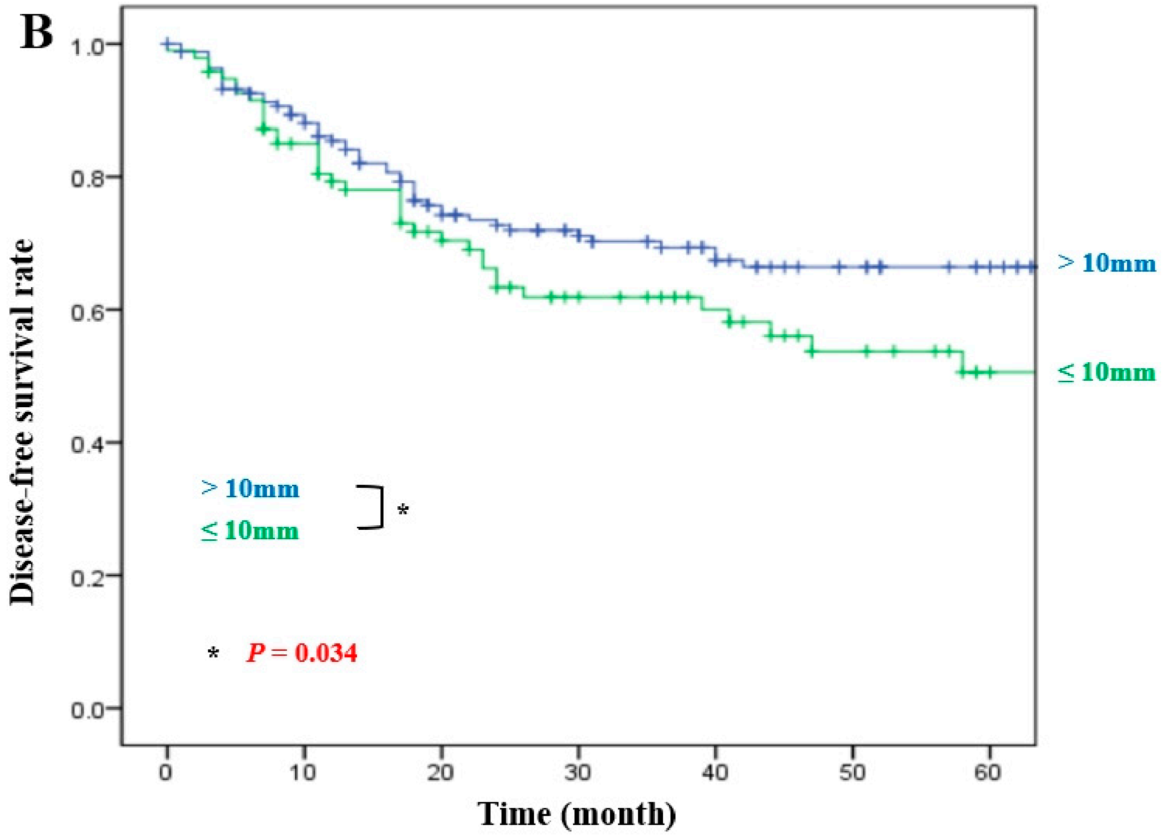

> 10mm	164	137	103	87	72	59	50
≤ 10mm	95	75	53	39	32	21	13

SUPPLEMENTARY FIGURE 1C

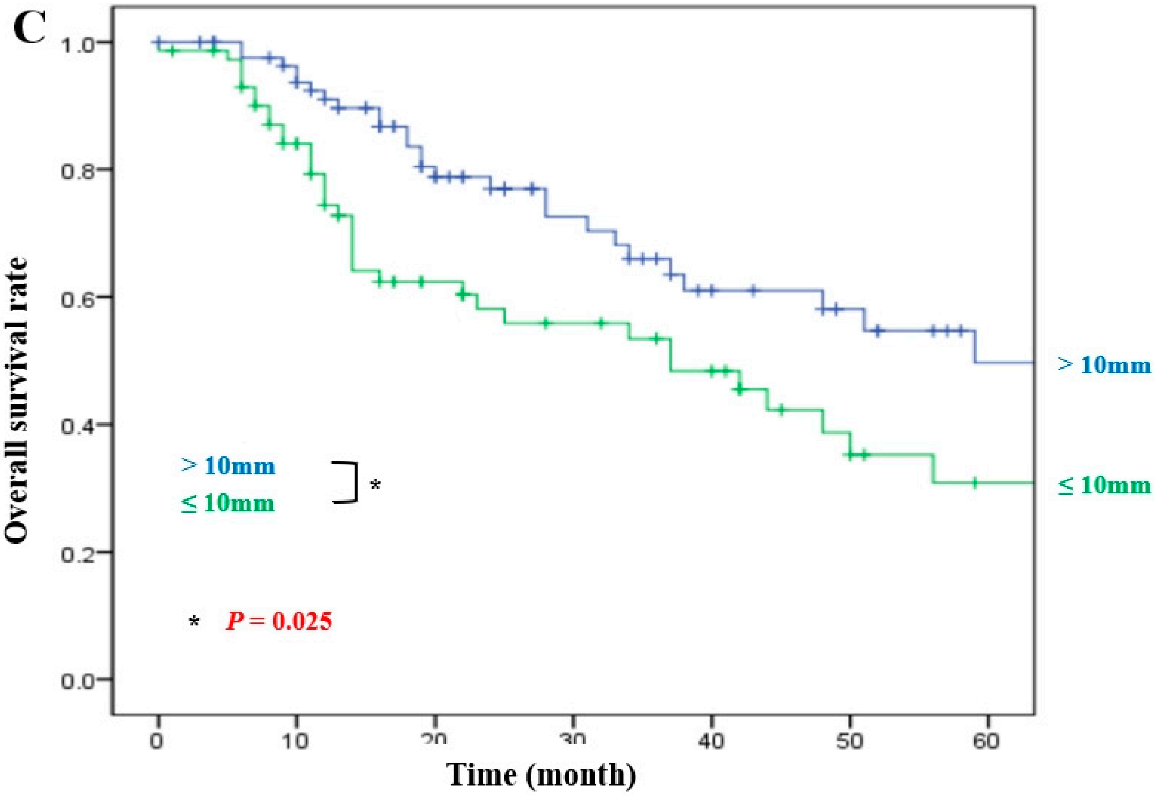

> 10mm	85	75	49	33	23	17	10
≤ 10mm	72	55	31	24	19	11	6

SUPPLEMENTARY FIGURE 1D

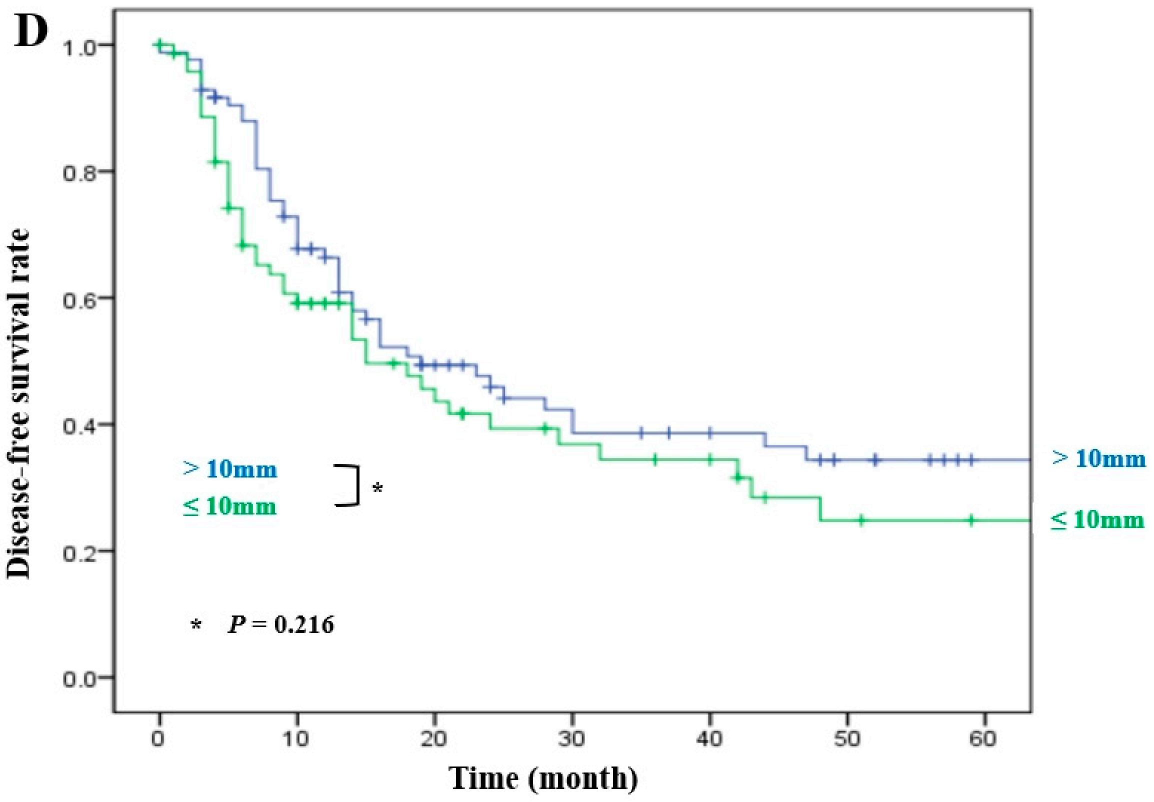

> 10mm	85	57	32	23	19	13	7
≤ 10mm	72	40	23	15	13	7	5

Figure S1. Comparison of (A) overall survival and (B) disease-free survival rates according to the ductal margin distance in patients without lymph node metastasis. Comparison of (C) overall survival and (D) disease-free survival rates according to the ductal margin distance in patients with lymph node metastasis.
